# Diagnostic accuracy of CompCog: reaction time as a screening measure for mild cognitive impairment

**DOI:** 10.1590/0004-282X-ANP-2021-0099

**Published:** 2022-08-08

**Authors:** Larissa Hartle, Marina Martorelli, Giulia Balboni, Raquel Souza, Helenice Charchat-Fichman

**Affiliations:** 1 Pontifícia Universidade Católica do Rio de Janeiro, Departamento de Psicologia, Rio de Janeiro RJ, Brazil.; 2 Università degli Studi di Perugia, Dipartimento di Filosofia, scienze sociali, umane e della formazione, Perugia, Italia.

**Keywords:** Cognitive Dysfunction, Reaction Time, Diagnosis, Dementia, Cognitive Aging, Disfunção Cognitiva, Tempo de Reação, Diagnóstico, Demência, Envelhecimento Cognitivo

## Abstract

**Background:**

Reaction time is affected under different neurological conditions but has not been much investigated considering all types of mild cognitive impairment (MCI).

**Objective:**

This study investigated the diagnostic accuracy of CompCog, a computerized cognitive screening battery focusing on reaction time measurements.

**Methods:**

A sample of 52 older adults underwent neuropsychological assessments, including CompCog, and medical appointments, to be classified as a control group or be diagnosed with MCI. The accuracy of CompCog for distinguishing between the two groups was calculated.

**Results:**

The results from diagnostic accuracy analyses showed that the AUCs of ROC curves were as high as 0.915 (CI 0.837-0.993). The subtest with the highest sensitivity and specificity (choice reaction time subtest) had 91.7% sensitivity and 89.3% specificity. The logistic regression final model correctly classified 92.3% of individuals, with 92.9% specificity and 91.7% sensitivity, and included only four variables from different subtests.

**Conclusions:**

In summary, the study showed that reaction time assessed through CompCog is a good screening measure to differentiate between normal aging and MCI. Reaction time measurements in milliseconds were more accurate than correct answers. This test can form part of routine clinical tests to achieve the objectives of screening for MCI, indicating further procedures for investigation and diagnosis and planning interventions.

## RESUMO

**Antecedentes:**

O tempo de reação é afetado em diferentes condições neurológicas, mas não foi muito investigado considerando todos os tipos de comprometimento cognitivo leve (CCL).

**Objetivo:**

Este estudo investigou a acurácia diagnóstica do CompCog, uma bateria computadorizada de rastreio cognitivo focada em medidas de tempo de reação.

**Métodos:**

Uma amostra de 52 idosos passou por uma avaliação neuropsicológica, incluindo o CompCog, e uma consulta médica para serem classificados como grupo controle ou serem diagnósticos com CCL. A acurácia do teste para distinguir entre os dois grupos foi calculada.

**Resultados:**

Os resultados das análises de acurácia diagnóstica mostraram AUC das curvas ROC tão altas quanto 0,915 (CI 0,837-0,993). O subteste com maior sensibilidade e especificidade - subteste de tempo de reação de escolha - apresentou sensibilidade de 91,7% e especificidade de 89,3%. O modelo final de regressão logística classificou corretamente 92,3% dos indivíduos, com especificidade de 92,9% e sensibilidade de 91,7%, e incluiu apenas 4 variáveis ​​de diferentes subtestes.

**Conclusões:**

Em resumo, o estudo mostrou que o tempo de reação avaliado pelo CompCog é uma boa medida de rastreio para diferenciar entre envelhecimento normal e CCL. Medidas de tempo de reação em milissegundos se mostraram melhores que o número de respostas corretas. O teste pode fazer parte de testes clínicos de rotina para atingir o objetivo de rastrear o CCL, indicar outros procedimentos para investigação e diagnóstico e planejar intervenções.

## INTRODUCTION

The American Academy of Neurology has acknowledged the utility of diagnosing mild cognitive impairment (MCI) as proposed by Petersen^1^. Its utility is related to the higher rate of conversion of individuals diagnosed with MCI to dementia than among those not diagnosed^2^, and to the possibility of implementing early interventions to improve quality of life^3^ ^,^ ^4^. 

The diagnosis of MCI has evolved over the years. Today, it includes subtypes with different etiologies and prognostics^5^. Thus, it is a heterogeneous construct that can involve subtle cognitive impairment of several functions that are not consistently detectable through commonly used screening tests^6^. It is a challenge to detect MCI in its early years, before it has progressed to severer forms of cognitive decline like dementia. Although research is making progress, it is usually more focused on (1) forms of MCI related to Alzheimer’s disease^7^ ^,^ ^8^ and (2) techniques using technologies that are not always accessible or used in screening processes, like neuroimaging and biomarkers^9^ ^,^ ^10^.

Evidence suggests that assessment of some cognitive variables may constitute a noninvasive and affordable first step regarding screening for cognitive decline^11^ ^,^ ^12^. One cognitive variable that might be affected more homogenously through MCI heterogeneity is processing speed. This can be assessed through several different variables^13^. Response speed and reaction time are the ones most used and can be understood as the time taken by an individual to issue a response after a stimulus^14^. This can be measured as a mean or median from several trials or also by considering intraindividual variability^14^. Although the findings have not been consistent, variability and reaction time are not necessarily impaired or affected simultaneously or with the same severity^14^. Nevertheless, concerning MCI, both measurements are of interest, given that studies have shown that both of them may be impaired^15^ ^,^ ^16^.

For a long time, processing speed has been seen as a fundamental aspect of cognition and an essential aspect of healthy aging^17^. Studies have shown that it declines through many neurodegenerative conditions^13^ ^,^ ^15^ ^,^ ^16^ ^,^ ^18^ ^-^ ^20^. One metanalysis found slower reaction time among MCI patients than among healthily aging individuals^21^. Although the majority of such studies only considered amnestic patients, the same results were found in two studies that also considered non-amnestic MCI patients^22^ ^,^ ^23^. 

Studies have shown that reaction time can decrease before errors start to be committed. Sometimes, a task can be completed, but in more time than usual^24^ ^,^ ^25^. However, this variable is not commonly measured, considering that (1) precision is required for detecting changes at the beginning of pathological aging processes^19^ ^,^ ^23^ and (2) paper-and-pencil neuropsychological assessment predominates^26^. Paper-and-pencil neuropsychological tests rarely involve precise reaction time measurements that can detect the subtle changes in the first stages of pathological aging^27^ ^,^ ^28^. However, one option for addressing this matter is to use computerized tests. These can be successful in this task because they provide precise reaction time measurements^29^. 

Use of computerized tests also brings other benefits, such as greater control over the administration and scoring of tests, reduction of errors in scoring and reduction of examiner’s bias^26^. This is especially true for low and middle-income countries, where resources are limited and there is a need for fast and cheap methods that are amenable to large-scale administration^10^. 

In the present study, we investigated whether the reaction time measurements of the CompCog computerized battery are helpful for discriminating between MCI and healthy individuals. This battery uses an iPad interface, and all responses are issued using a touchscreen. During each test, the type of response and reaction time in milliseconds are recorded. A previous version of the same test is already known to distinguish between healthy individuals and individuals with Alzheimer's disease^30^. Thus, CompCog was expected to form a valuable tool for detecting MCI.

## METHODS

### Setting and procedures

Participants were invited after involvement in a larger study conducted in partnership with a social program offered by Rio de Janeiro's government^31^. This program provides daily activities for older adults during the day, such as physical exercises, stretching, yoga, dance, cognitive stimulation, crafts, theater, etc. The first study evaluated older adults through a brief neuropsychological assessment done by researchers and senior neuropsychologists. All psychologists attended weekly supervision with the coordinator of the Applied Psychology Service of the Pontifical Catholic University of Rio de Janeiro. The evaluation lasted one hour and was held in a quiet room in the houses where the social program commonly took place. During the assessment, cognitive tests and scales were used to assess cognition, depressive symptoms and functionality. These are all described in the corresponding section below.

Participants in the larger study were randomly invited to join the present study. The ones who accepted this underwent another neuropsychological testing session and a medical appointment with a doctor, at which diagnoses were given. The neuropsychological assessment consisted of (1) a new anamnesis to confirm the clinical and sociodemographic characteristics of the individuals, and the inclusion and exclusion criteria for recruitment; and (2) administration of CompCog. The average session duration was 1h15, and the sessions were carried out at the Applied Psychology Service of the Pontifical Catholic University of Rio de Janeiro. Medical appointments aimed at making diagnoses were conducted at the same place or in the outpatient clinics of the Department of Medicine of the same university.

Geriatricians evaluated the cases and made the diagnoses during medical appointments. The diagnoses were based on clinical history, neuroimaging when available and the initial neuropsychological protocol. This protocol included the following tests and scales: 1) Mini-Mental State Examination (MMSE)^32^; 2) Brief Cognitive Screening Battery^31^ ^,^ ^33^ consisting of the following tests: Figure Memory Test (MFT), Categorical Verbal Fluency Test (VF) and Clock Drawing Test (CDT); 3) Geriatric Depression Scale (GDS-15)^34^; 4) Functional Activities Questionnaire (FAQ)^35^ and 5) Lawton Instrumental Activities of Daily Living Scale^36^. Although the FAQ formed part of the evaluation, it was not used in the analysis because of a high rate of missing data. The maximum interval between the first evaluation and the medical appointment was six months.

### Participants

Seventy older adults (above 60 years old) were recruited for this study. Among them, 40 were classified as healthy older adults, i.e. individuals with no changes in cognitive performance tests and without functional impairment. The other 30 were diagnosed as older adults with MCI. Exclusion criteria eliminated six individuals from the MCI group and two individuals from the control group (CG). The exclusion criteria were the following: (1) presentation of conditions other than MCI that affect cognition (e.g. stroke); (2) recent history of alcohol or other drug dependence; (3) high levels of depressive symptoms, assessed from the score on the depression scale; (4) presence of visual or hearing disorders without correction; (5) illiteracy; and/or (6) use of medications that could affect reaction time (e.g. benzodiazepines). In the CG, 10 cases were randomly excluded until the variables of number of years of education, sex, age, number of health issues, depressive symptoms and number of medications in use had become matched with those of individuals in the MCI group. The resulting sample consisted of 24 participants with MCI and 28 individuals in the CG. The mean age of the MCI group was 73.9 years (6.9); the mean number of years of education was 11.6 (5.3); and 70.8% were women. The mean age of the CG was 71.4 years (5.7); the mean number of years of education was 14.1 (3.3); and 82.1% were women.

Although the diagnosis did not include the MCI type, it was possible to propose a classification into amnestic or non-amnestic based on the paper-and-pencil tests used, i.e. the tests in the Brief Cognitive Screening Battery. Out of the 24 MCI participants, 13 had at least one Z score below -1 in the memory test, and therefore these individuals could be classified as presenting an amnestic MCI type. The other 11 participants did not have Z scores below -1 in the memory test, and therefore could be classified as presenting a non-amnestic MCI type. We consider that these data were insufficient to classify the amnestic or non-amnestic types as multi-domain or single-domain, because more extensive assessments might have shown more deficits^37^. Nevertheless, the differences between the control group and the MCI group are described in the results. 

### Instrument

CompCog is a computerized cognitive screening battery with eight subtests that evaluate different cognitive domains: Simple Reaction Time (SRT), Choice Reaction Time (CRT), Implicit Learning Test (ILT), Visual and Spatial Short-Term Memory (STM), Face Recognition and Memory (FRM), Inhibitory Control Test (ICT), Stroop Test (StT) and Survey Test (ST). The subtests are usually presented in this order but can also be randomized. In our study, we used the standard test order. Each subtest is explained in Table 1 with the respective variables evaluated (52 in total). All responses are issued using a touch screen and recorded. All tests generate reaction time measurements registered in milliseconds for each touch and are presented as the total time and median time, in order to eliminate possible discrepant data from each test.

**Table 1.** CompCog tests and variables.

Test	Cognitive functions involved and how they are evaluated	Variables
Simple Reaction Time (SRT)	Processing speed. As soon as a white square appears in the middle of the screen, the person should touch the rectangle at the bottom of the screen.	Median reaction time
Choice Reaction Time (CRT)	Processing speed. As a white or orange square appears in the middle of the screen, the person should touch the rectangle of the same color at the bottom of the screen.	Median reaction time; Correct responses; Revised median reaction time (choice reaction time - simple reaction time).
Implicit Learning Test (ILT)	Implicit learning. As one of ten gray squares distributed in the screen turns white, the person should press it. There is a fixed sequence of 25 squares that is repeated four times and one last random sequence.	Median reaction time in each of five tasks; Implicit learning (median reaction time in sequence 4/median reaction time in sequence 1).
Visual and Spatial Short-Term Memory (STM)	Working memory. There are ten gray squares distributed on the screen. One will become white at a time, making a sequence that should be reproduced.	Correct responses; Direct order SPAN; Median reaction time in direct order; Inverse order SPAN; Median reaction time in inverse order.
Face Recognition and Memory (FRM)	Episodic memory. Ten drawings of unknown faces are presented for 30 seconds. The participant should then choose from among ten pairs of faces, the one that was among those initially shown for memorization, in four attempts.	Correct responses and median reaction time for each of the four tasks and for all tasks together.
Inhibitory Control Test (ICT)	Attention and inhibitory control. Squares of different colors will appear in the middle of the screen for one second each: the white ones should be avoided.	Median reaction time; Correct responses; Median reaction time for correct responses; Median reaction time for errors; Errors.
Stroop Test (StT)	Attention and inhibitory control. All tasks have four colored rectangles located at the bottom of the screen. The person should touch the one matching the stimulus that appears in the middle of the screen considering its color without distracters (task 1) and with distracters (tasks 2 and 3).	Interference; Median reaction time and errors for each of the three tasks.
Survey Test (ST)	Attention. Squares of different colors will appear in the middle of the screen for one second each. Participants should press the white ones in the first task, whites and blues in the second and also yellow ones in the third.	Median reaction time, correct responses, reaction time for correct responses, errors and reaction time for errors, for each of the three tasks.

Furthermore, correct response percentages, errors and differences in reaction time between errors and correct responses are also registered. All the stimulus tests are visuospatial, except for one test: the Stroop Test, which contains written words to maintain the original paradigm^38^. With two exceptions, all reaction time medians are calculated after more than 50 trials, with a maximum of 100 trials. The FRM test has a total of 40 trials total, and STM test trials depend on correct responses, with a maximum of 105 trials. 

A previous version of the same test is already known to distinguish between healthy individuals and individuals with Alzheimer's disease^30^. Previous analyses regarding the current version showed (1) good construct validity in a principal component analysis, in which variables clustered in agreement with the subtest divisions; and (2) good concurrent validity, with moderate and strong correlations between the CompCog tasks and their equivalents in paper-and-pencil tests^39^.

### Ethics

The National Commission for Research Ethics approved this study (opinion no. 965.264; CAAE: 39381514.3.0000.5285) through the UNIRIO Research Ethics Committee. Individuals participated in the study through signing a free and informed consent statement that had been drawn up in accordance with resolution 196/96 of Brazil's National Health Council, which deals with guidelines and standards for research involving human individuals. Participation in this survey was voluntary and the participants did not receive any payment. The study did not bring any risk to the participants' health and they could refuse and/or withdraw consent to participate in the study at any time.

### Statistical analysis

All analyses were conducted using the Statistical Package for the Social Sciences (SPSS, version 22). After verifying through Shapiro-Wilk tests whether the data were normally distributed, differences between groups were tested using t tests for normal distributions or Mann-Whitney tests for non-normal distributions. A chi-square test was used in the case of sex. Receiver operating characteristic (ROC) analysis was performed for each CompCog variable. ROC curves were plotted in order to determine the degree to which subtests discriminated between controls and MCI. As proposed in a recent meta-analysis^6^, sensitivity was prioritized instead of specificity since we were offering a screening measure. Therefore, false positives would be better than false negatives, with regard to continuing the clinical investigation. This prioritization was done by choosing the highest sensitivity that still allowed specificity of at least 70%. This method could not be followed regarding five variables for which specificity of at least 70% would cause sensitivity lower than 70%. In those cases, the cutoff point with sensitivity higher than 70% for which the specificity was closest to 70% was chosen. 

 The variables with higher sensitivity and specificity in ROC analyses were then used in a logistic regression model with the stepwise forward method, to create a model for predicting MCI with the least number of variables. All variables with specificity and sensitivity above 70% were included (24 variables in total). Age and the number of years of education were also included in order to ascertain whether they influenced the model. 

## RESULTS

### Sample characteristics

The participants’ performance in neuropsychological assessments and their demographic and clinical characteristics are described in Table 2. There was a tendency towards no significant difference between the groups regarding educational level in years (*t*(37.486) = 2.008; *p* = 0.052), and there were no significant differences regarding age (*t*(50) = -1.414; *p* = 0.164), number of health problems (*t*(40.60) = -0.0846; *p* = 0.403), number of medications in use (*t*(50) = 0.203; *p* = 0.840), number of depressive symptoms (*t*(50) = -1.234; *p* = 0.224) and sex (*x²* (1) = 0.931; *p* = 0.335).

**Table 2.** Clinical characteristics of the sample.

Variable	CG (n = 28) Mean (SD), min-max	MCI (n = 24) Mean (SD), min-max	p-value
Sex*	23/5	17/7	0.335
Age	71.4 (5.7), 62-83	73.9 (6.9), 61-85	0.164
Number of years of education	14.1 (3.3), 6-17	11.6 (5.3), 3-18	0.052
Health problems	1.5 (1.0), 0-3	1.7 (1.4), 0-4	0.403
Medications in use	2.0 (1.9), 0-6	1.9 (2.0), 0-6	0.840
Depressive symptoms	2.8 (1.9), 0-6	3.6 (2.6), 0-9	0.224
Naming	10.0 (0.0), 10-10	9.8 (4.4), 8-10	0.123
Incidental memory	5.9 (1.2), 4-8	5.2 (1.5), 3-8	0.084
Immediate memory**	8.5 (1.1), 6-10	7.2 (1.4), 5-10	0.001
Learning**	9.3 (0.8), 8-10	8.3 (1.1), 6-10	0.001
Delayed recall**	8.6 (1.5), 4-10	7.2 (1.6), 4-10	0.005
Recognition	9.7 (0.4), 9-10	9.5 (0.9), 7-10	0.930
Clock drawing test	6.2 (2.2), 4-10	5.5 (2.5), 1-10	0.259
Verbal fluency**	20.7 (4.3), 13-30	16.6 (4.6), 8-26	0.002
MMSE30	27.0 (2.1), 22-30	25.7 (2.7), 21-29	0.067
Functionality**	20.9 (0.2), 20-21	19.6 (1.6), 15-21	< 0.001

* # female/male; ** significant differences between groups.

There were differences in cognition with regard to immediate memory (*t*(50) = 3.562; *p* = 0.001), learning (*t*(50) = 3.572; *p* = 0.001), delayed recall (*t*(50) = 2.914; *p* = 0.005) and verbal fluency (*t*(50) = 0.732; *p* = 0.002). Functionality also differed between the groups (*U* = 1440; *Z* = -4.257; *p* < 0.001). The tests did not show differences regarding naming (*U* = 308; *Z* = -1,542; *p* = 0.123), incidental memory (*t*(50) = -1.764; *p* = 0.084), recognition (*U* = 332.5; *Z* = -0.087; *p* = 0.930), clock drawing test (*t*(50) = 1.141; *p* = 0.259), and MMSE (*t*(50) = 1.873; *p* = 0.067).

*ROC curve*

The area under the ROC curve (AUC) for all variables can be seen in Table 3. For significant variables, the same table shows sensitivity, specificity and cutoff points. 

**Table 3.** AUC for all variables; cutoff points, sensitivity and specificity for significant variables.

Test	AUC, 95% confidence interval range	Cutoff point (milliseconds)	Sensitivity/specificity
Simple Reaction Time Test			
MRT	0.506, 0.339-0.673		
Choice Reaction Time Test			
MRT†	0.915, 0.837-0.993*	689.813	91.7%/89.3%
Correct responses	0.650, 0.498-0.801		
Revised MRT	0.705, 0.559-0.852		
Implicit Learning Test			
MRT 1	0.839, 0.721-0.957*	688.125	83.3%/75%
MRT 2	0.836, 0.720-0.953*	651.531	75%/75%
MRT 3	0.823, 0.703-0.943*	616.159	79.2%/67.9%
MRT 4	0.829, 0.710-0.948*	618.139	75%/71.4%
MRT 5	0.804, 0.674-0.933*	664.784	75%/75%
Implicit learning	0.521, 0.361-0.681		
Visual and Spatial Short-Term Memory Test			
Correct responses	0.718, 0.577-0.859		
Direct order SPAN	0.725, 0.588-0.861		
MRT in direct order	0.774, 0.642-0.906*	643.635	75%/71.4%
Inverse order SPAN	0.868, 0.774-0.962*	3.5	95.8%/64.3%
MRT in inverse order	0.781, 0.648-0.915*	668.615	75%/67.9%
Face Recognition and Memory			
MRT†	0.896, 0.799-0.993*	1580.791	83.3%/85.7%
MRT 1	0.823, 0.703-0.943*	1905.385	75%/71.4%
MRT 2	0.881, 0.781-0.981*	1506.316	91.7%/75%
MRT 3	0.872, 0.767-0.977*	1430.946	87.5%/75%
MRT 4	0.813, 0.693-0.932*	1486.333	79.2%/71.4%
Correct responses	0.811, 0.685-0.937*	98.750	79.2%/57.1%
Correct responses 1	0.763, 0.628-0.899		
Correct responses 2	0.673, 0.537-0.838		
Correct responses 3	0.688, 0.537-0.838		
Correct responses 4	0.757, 0.619-0.894		
Inhibitory Control Test			
MRT	0.884, 0.782-0.976*	664.447	87.5%/78.6%
CAMRT	0.871, 0.774-967*	663.279	83.3%/78.6%
EMRT	0.753, 0.615-0.891		
Correct responses	0.794, 0.664-0.924*	96.5	75%/75%
Errors	0.794, 0.664-0.924*	3.5	75%/75%
Stroop Test			
MRT 1	0.847, 0.732-0.962*	814.839	87.5%/75%
MRT 2	0.799, 0.672-0.926*	911.100	83.3%/67.9%
MRT 3	0.743, 0.604-0.881		
Errors 1	0.587, 0.430-0.744		
Errors 2	0.541, 0.378-0.704		
Errors 3	0.525, 0.336-0.684		
Interference	0.506, 0.343-0.669		
Survey Test			
MRT 1	0.818, 0.703-0.934*	637.844	79.2%/71.4%
Correct responses 1	0.648, 0.497-0.799		
CAMRT 1	0.818, 0.703-0.934*	637.844	79.2%/71.4%
Errors 1	0.648, 0.497-0.799		
EMRT 1	0.465, 0.307-0.623		
MRT 2	0.829, 0.714-0.944*	663.004	79.2%/75%
Correct responses 2	0.798, 0.676-0.919*	97	79.2%/71.4%
CAMRT 2	0.835, 0.721-0.949*	663.004	79.2%/75%
Errors 2	0.798, 0.676-0.919*	3	79.2%/71.4%
EMRT 2	0.573, 0.415-0.731		
MRT 3	0.823, 0.707-0.939*	653.629	83.3%/71.4%
Correct responses 3	0.802, 0.678-0.926*	93	75%/75%
CAMRT 3	0.826, 0.711-0.941*	664.223	83.3%/75%
Errors 3	0.802, 0.678-0.926*	7	75%/75%
EMRT 3	0.507, 0.346-0.669		

MRT: median reaction time; AUC: area under the ROC curve; CAMRT: median reaction time for correct response; EMRT: median reaction time for error; †best accuracy; *p < 0.001.

In general, reaction time measurements in cognitive tasks of lower complexity (e.g. choosing between colors) and memory tasks were the variables that best discriminated between the CG and MCI group. Simple reaction time, reaction time relating to the Stroop effect, reaction time regarding errors, number of errors and number of correct responses did not differentiate between the CG and MCI group. 

### Regression models

The final model correctly classified 92.3% of the individuals, with 92.9% specificity and 91.7% sensitivity, and included four variables. All of these variables concerned reaction time, but in four different tasks: the first task of the Stroop test (odds ratio = 0.979; 95% CI = 0.963-0.996; *p* = 0.015); the inhibitory control test (odds ratio = 1.027; 95% CI = 1.007-1.048; *p* = 0.008); the second task of the memory test (odds ratio = 1.009; 95% CI = 1.001-1.017; *p* = 0.021); and the second sequence of the implicit learning test (odds ratio = 1.018; 95% CI = 1.001-1.036; *p* = 0.033). Age and the number of years of education did not influence the model. The final model had a chi-square value of 46.183 (4); *p* < 0.001. The -2 log likelihood was 25.597, with Cox & Snell R-square of 0.589 and Nagelkerke R of 0.786.

## DISCUSSION

### Differences between paper-and-pencil and computerized tests

The first thing to notice is the neuropsychological profile of the sample. Significant differences were found between the groups in paper-and-pencil tests evaluating episodic memory and semantic verbal fluency. The latter has been reported to be highly dependent on semantic memory^40^. Episodic and semantic memory impairments are characteristics of the amnestic subtype of MCI^41^. On the other hand, the reaction times in CompCog tasks involving memory, attention and executive functions showed good accuracy in distinguishing between participants with MCI and the CG. 

These results suggest that there is a potential benefit from using computerized tests. These can track a more significant number of impairments than those typically measured through traditional paper-and-pencil assessments. Moreover, with regard specifically to memory performance in CompCog, the accuracy of the number of correct responses was not as high as that of the reaction time. 

There are differences between the CompCog memory task and the paper-and-pencil memory test. The CompCog task uses recognition and not recall, as the paper-and-pencil test does. This difference suggests that the CompCog task is easier. 

Two benefits can be extracted from this information. The first is the possibility of evaluation without generating performance anxiety and frustration^42^, since the numbers of correct responses are similar between the groups. The second is the ability to distinguish between groups before errors start to be committed. 

One hypothesis in this regard is that a slower reaction time is one of the first cues of cognitive impairment. Other studies have already shown that the time required for completing tasks increases^25^, even before errors hinder their completion^24^. There is also evidence of a correlation between reductions in processing speed and general cognitive performance.^18^ It is interesting to note that a reduction in processing speed is also related to subjective memory complaints^43^. Although this kind of complaint usually does not involve an objective deficit in standard tests, it is possible that patients somehow already perceive their slower reaction time. A meta-analysis has suggested that people with subjective memory complaints have twice as high a risk of developing MCI and dementia as do older adults who have no complaints^44^. However, their condition is difficult to measure through traditional memory tasks because individual performances are similar to those of controls^45^.

### ROC curve: reaction time is useful as a screening measure for MCI

In general, the ROC curve results showed that reaction time measurements on different cognitive processes were good at distinguishing between healthy individuals and participants with MCI. In comparing these measurements with the numbers of errors and correct responses in the same subtest, the sensitivity and specificity of the reaction time were usually higher, considering reaction times. Normal aging is known to correlate with slower reaction time^13^ ^,^ ^18^. However, the results showed signs that the decline might be even more considerable under certain circumstances of pathological conditions, such as in relation to cognitive processes of low and moderate complexity. This conclusion can be drawn from numerous results, but a comparison between the first two subtests might be the clearest: (1) simple reaction time, which was not good at distinguishing the groups; and (2) choice reaction time, which showed the best accuracy, with AUC as high as 0.9. 

The results in the literature regarding the topic are mixed. Some studies investigated reaction time in simple tasks and found that this showed good accuracy for distinguishing between participants with MCI and controls^46^ ^-^ ^48^. In one study^49^, the effect of increasing complexity stimulus was investigated and a division of reaction time into a movement component and a cognitive component was proposed. Activities that solely involved motor reactions, without decision making, could be used to differentiate between patients with Alzheimer’s disease and cognitively healthy old adults, but not between the latter and MCI patients. Only the cognitive component was sensitive to MCI, which suggests that although lower complexity tasks may be useful in this regard, at least some cognitive processing must be involved. This may explain why the Simple Reaction Time test (motor component only) could not distinguish between the groups, but the Choice Reaction Time could, which is a low-complexity cognitive component. 

Nevertheless, the same study^49^ and others^19^ ^,^ ^50^ found that more complex variables were better at distinguishing between groups, i.e. a contrary finding. One hypothesis for these contrasting results is that these studies used only the amnestic subtype of MCI. Using just one subtype creates a more homogeneous sample concerning cognitive impairment. So, perhaps, using more subtypes would produce different results. For example, cognitive impairment in complex cognitive processes would be more heterogeneous, and reaction time in simple cognitive tasks would still be homogenously impaired in the sample.

 Another common problem in research that may cause divergence is how reaction time is measured and reported. Some studies have suggested that intraindividual variability is higher in individuals going through cognitive decline and, therefore, in patients with MCI^49^ ^,^ ^51^. Although measurement of intraindividual variability itself can be worth investigating, it can create noise when the goal is to compare reaction time. Mean results from one or a few trials might not provide a good comparison measurement. CompCog does not have this problem since it uses the median reaction time derived from multiple trials. This would eliminate the variability problem that affects the MCI sample and does not affect the control sample. Even so, studying the intraindividual variability itself is another option for future studies with CompCog.

In addition to the abovementioned benefits of some computerized tests, two more can be added in the same context. First, simple choice reaction time can be evaluated longitudinally and without a learning effect. This enables longitudinal follow-up in which individuals will be compared with themselves in order to detect any decline right from its beginning, with the consequent possibility of early interventions.

Lastly, comparison between the reaction times for errors and correct responses in the two subtests that measure it (Survey test and Inhibitory Control test) showed that only the reaction times for correct responses could differentiate between the groups. Separated variables showing reaction times for errors and correct responses are not common in tests. The majority of computerized tests still use the same measurements used in paper-and-pencil tests, i.e. errors and total scores. The tests that investigate reaction time mainly focus on attention processes^52^ ^,^ ^53^, probably because the cognitive process construct is highly relatable to processing speed^23^. However, the results show that reaction times are not the same between situations of getting answers right or wrong. These differences might be worth considering as variables if new tests are created and might be worth investigating in future studies. 

### Regression models

The final model that best predicted MCI with the least number of variables included three reaction time measurements regarding attention and one regarding memory, which correctly classified 92.3% of the individuals. The direction of the reaction times in the inhibitory control test, the second task of the memory test and the second sequence of the implicit learning test differed from the direction of the fourth variable selected, i.e. the reaction time in the first task of the Stroop test. Upon closer inspection, we hypothesized that the MCI group committed more errors, while the healthy group took more time in order to avoid mistakes. 

We propose that these results should be seen as an exploratory analysis. It could be difficult to use only the selected variables in a test, because variables inside tests from unrelated tasks were selected for the model. Even so, the model suggests that a reaction time score composed of performance levels in different tasks could have even higher accuracy than reaction time measured separately. This proposal has to go through further testing in future studies with a specific hypothesis and larger samples.

In conclusion, we can infer from the results that reaction time measurements through CompCog are an efficient and accurate way to screen for MCI. Although the initial cost of the equipment might be high, there is no maintenance cost for its administration thereafter. There is also the possibility of expanding the technology to other devices in future studies, such as to cellphones. Thus, this method could form a low-cost option for screening for MCI on a large scale. Low-cost options are especially necessary in low and middle-income countries^54^. It is not our proposal to use the test as a diagnostic tool but to bring in technology that allows doctors or caregivers to perform simple screening on individuals who are at the threshold of old age. Additional tests and investigations should be done to reach a diagnosis and indicate treatments, depending on the results. 

In order to achieve the above objective, more evidence needs to be produced. To assess cognitive decline, it is important to compare individuals with themselves at different times^9^, which is a matter that our study could not cover. The best way to screen for MCI would be to compare individuals' results year by year. Studies with follow-up could provide more evidence of the utility of CompCog for MCI screening. 

Furthermore, two other variables that could have been controlled for were the individuals’ subjective cognitive decline and the time that elapsed between the first evaluation and the diagnosis. Controlling for the latter could have ensured that the length of time between the diagnosis and the neuropsychological assessment did not influence results. Controlling for subjective cognitive decline could have shown how and whether reaction time relates to cognitive complaints. 

Lastly, the sample size can also be seen as a limitation of the present study. Although there is a need for larger samples to achieve more reliable results, there is a lack of studies exploring all MCI subtypes together. Most studies have explored Alzheimer's disease and amnestic MCI. Other MCI subtypes have been less investigated and, therefore, our findings remain relevant. Our results show that CompCog is a useful tool for screening for cognitive impairment regardless of the etiology, with reaction time measurements that are easy to obtain. CompCog can be a practical and advantageous instrument for selecting patients for a more comprehensive neuropsychological assessment and, therefore, enabling early diagnosis of MCI. 
